# Supplying Relief: Civil Medical Assistance during the Korean War, 1950–3

**DOI:** 10.1093/shm/hkaf068

**Published:** 2025-08-19

**Authors:** Dongkue Lee, Mark Harrison

**Affiliations:** * The HK+ Institute for Integrated Medical Humanities, Kyung Hee University, Seoul, South Korea

**Keywords:** Korean war, medical relief, UNKRA, United Nations, medical supply

## Abstract

This study examines the United Nation (UN)’s efforts to provide emergency relief for civilians during the Korean War, one of the deadliest and most devastating conflicts for civilians since World War II. The UN coordinated civilian relief during and after the war, developing a logistical system, legal framework and payment mechanism through UK banks in pounds sterling, and a distribution strategy based on European experience. United Nations Korean Reconstruction Agency (UNKRA) managed financial and legal issues, determined packing and shipping methods, resolved storage problems and distributed medical supplies in the field, either through United Nations Civil Assistance Corps Korea or UNKRA. The UN’s efforts to provide civilian medical assistance to Korea set an exemplary model for later relief efforts and this study shows how this holistic system was created on the basis of experience gained during World War II and the creation of entirely new mechanisms for purchasing and delivering medical aid.

## Introduction

The Korean War was the first international conflict fully involving the United Nations (UN), which was established after World War II. While the US armed forces directed most of the military operations, the UN also implemented a civilian rehabilitation programme during and after the armistice. This initiative included a new supply chain and a long-term rehabilitation programme, leading to the distribution of medical supplies and the establishment of a hospital system.^1^ When the civilian situation in South Korea became medically difficult, one of the most basic and urgent tasks was establishing a secure supply chain.^2^ While logistics have always been an essential aspect of warfare, the planning and maintenance of supplies increasingly depended on coordination between civil and military domains.^3^ This study traces the medical supplies provided by the UN for civilian relief during the Korean War, emphasising their significance in public health and medical relief efforts within the context of a large-scale emergency.^4^

The UN’s efforts to provide civilian medical assistance offer new insights into its role in building Korean society—a society utterly devastated by the conflict of 1950–3^5^—and the mechanisms of civil assistance that operated in later UN interventions. The efforts had a precursor named the United Nations Relief and Rehabilitation Administration (UNRRA), formally established in 1943, a year and a half before the UN itself was founded, and it had discontinued operation by mid-1947.^6^ UNRRA brought international collaboration to civilian relief in Europe after World War II. Throughout UNRRA’s brief existence, relief planners recognised the importance of careful planning, coordination and laying the groundwork for long-term economic reconstruction to prevent disastrous results.^7^ The experience of post-war planning for ‘a new world order’ solidified UNRRA, which was quickly followed by a vast network of additional international and transnational efforts.^8^ Unlike previous civilian aid efforts, including UNRRA, which were generally temporary and reliant on public and private charity, the UN-led system aimed for comprehensive collective security and long-term rehabilitation for socio-economic challenges beyond emergency relief.^9^

This article examines the system for procurement of medical drugs and supplies for civilian medical relief by the UN, analysing documents relating to United Nations Korean Reconstruction Agency (UNKRA) in the UN Archives and United Nations Civil Assistance Corps Korea (UNCACK) in the National Archives of Korea and the USA.^10^ The UNKRA entered Korea in 1951, assigned liaison and planning duties with the possibility of a secondary executive position under the United Nations Command (UNC). However, UNKRA was scheduled to begin taking full responsibility 180 days after the end of hostilities. UNKRA would be responsible for long-range planning and high-level technical assistance to the Korean government and for any programme of economic aid. UNKRA also held responsibility for all UN relief and rehabilitation activities in agriculture, fisheries, education and industry.^11^ However, the long delay in the cessation of hostilities postponed the planned reconstruction, making the situation in Korea precarious.^12^ In the mean-time, primary responsibility remained with the UNC, operating though the UNCACK.^13^ Consequently, while the UNC managed both the military operation and civilian relief in South Korea, UNKRA collaborated with the UNC to procure medical supplies and establish a comprehensive plan to rehabilitate Korea post-war.^14^ Generally, under the United Nations General Assembly, UNKRA focussed on long-term operations, such as educating medical experts, building an infrastructure and establishing a nationwide medical system. Meanwhile under the commander-in-chief of the UNC, the UNCACK addressed imminent issues like controlling contagious diseases and distributing vaccines.^15^ This study highlights the collaboration between two organisations in civilian relief during wartime.

Comprehensive studies on both world wars have expanded our understanding of army mobilisation, the wartime supply chain and the logistics of transportation, evacuation and hospitalisation.^16^ Studies of logistics during the Korean War have provided essential insights into the US logistical system in the 1950s.^17^ However, most researchers have focussed on the US Army or UN forces more generally, with little attention having been paid to civilian relief driven by the UN. In addition, while UNCACK, under the UNC, was responsible for the actual distribution of medicines—a topic that is well-researched—it is less known that the UN headquarters and later UNKRA also acquired and supplied these medicines.^18^ Indeed, the medical logistics of most modern wars and attempts at post-war stabilisation have been barely examined. The UN developed a separate supply chain from the US market, including a logistical system, legal framework, payment through UK banks in pounds sterling and a distribution strategy based on European experience that was distinctly different from the USA-led military sections; however, in Korea, where the war was still ongoing, the UNC took the lead in distribution.^19^ Even after UNKRA finished its mission in 1958, these elements were integral to the initial UN system and played a crucial role in international politics in the following decades, particularly in the context of civilian relief efforts. In particular, this study focuses on the logistics of civil assistance, showing how an entirely new system had to be created for the purchase and delivery of medical commodities outside of military structures.^20^ In the field, the delivery of care employed templates derived from military or, at least, wartime experience, but the system as a whole had many new features, and it is these that this article accentuates. The article shows how the supply chain was constructed and attempts to overcome the various problems that occurred in obtaining and supplying the commodities that were so badly required in Korea. Without an effective means of delivering medical commodities, stabilisation of the health emergency in Korea and the rapid development of its medical system would have been impossible.

### Initial Response

By the mid-twentieth century, medical commodities were a vital part of relief efforts and public health campaigns more generally.^21^ During the Korean War, they were sometimes provided to civilians by UN forces, although this was typically a secondary task.^22^ Beyond the battle lines and away from military medical institutions, civilian relief was organised separately from military needs. When the Korean War broke out, Trygve Lie, a former Norwegian Minister of Foreign Affairs, was the first UN Secretary-General. Brock Chisholm, a Canadian psychiatrist, was the WHO’s director-general. Chisholm clearly mentioned that the WHO assisted the UN by providing health-related relief to the civilian population of Korea.^23^ The UN initiated its civilian relief efforts by collaborating directly with the WHO’s regional office in Washington, DC to procure medical supplies for civilian emergency relief. As with military operations, one of the most basic and urgent tasks was establishing a secure supply chain. UN Secretary-General Trygve Lie estimated potential costs, prepared lists of required items and procured medical supplies for civilian relief. This procedure included a market report, an order list for 29 items from August and another order list for 119 items from November.^24^

Responding effectively to the crisis in Korea required thorough research. The North-Strong Corporation, a private survey company, was engaged by the UN to prepare a report on the global medical market. The report, prepared for Fred L. Soper, a renowned American epidemiologist in charge of the WHO’s regional office in Washington, DC, was also distributed to the UN office.^25^ The report focussed on specific medical and surgical items in a commodity market that had been strained by the Korean conflict, as well as stockpiling due to both military needs and civilian demands. In many instances, producers and stockholders in the pharmaceutical industry created ‘artificial scarcity’ by withholding their inventory until the market settled.^26^ Suppliers also hedged against potential price increases in the coming months. Initially, the rate of price increases for sensitive items in the market was as high as 12–15 per cent per month. In this unstable environment, surging military and civilian demand tightened the market and drove up prices.^27^ The report expected that the price would level off within 2 or 3 weeks.

Medical commodities with military specifications were in shorter supply than identical commodities with non-military or commercial standards. This was particularly true for gauze and gauze bandage products. The report recommended that the UN should adopt a strategic purchasing approach to avoid triggering price hikes. This suggested placing a series of smaller orders with multiple providers rather than market-stimulating purchases. However, explosive wartime demand and the fact that most supplies had to be transported over long distances made this extremely challenging.^28^

Although the UN military response officially began 3 days after the war broke out, the UN’s civilian relief efforts did not commence until August—3 months into the conflict—with the shipment of 29 types of medical supplies ranging from blood products to vaccines and pharmaceuticals.^29^ This was in partial fulfilment of the UNC’s requests, specifically for civilian relief rather than military use.^30^ The UNC requested that the Secretary-General call for civilian relief in the form of urgently needed commodities, including medical supplies. The Secretary-General then identified member states and international organisations capable of providing these supplies, communicated the UNC’s list of requested items to the states and organisations and processed applications for assistance from those states. These applications were forwarded to the UNC, which worked directly or through UN affiliates with the relevant governments and organisations to determine the specifications of the supplies and negotiate the logistics of their delivery to South Korea. This coordination allowed the UN to organise assistance for Korea so that it could be handled efficiently through the UNC.^31^ However, in the case of the 29 medical items ordered in August, the WHO was responsible for purchasing medical supplies for public health teams, covering each provincial level in South Korea.^32^

Lacking in medical expertise, the UN relied on the WHO’s regional office for information regarding market conditions and how to secure supplies. This was not an easy task, as the requirements were very precise. For example, for medical use, alcohol needed to be ethyl, not methyl, and 95 per cent ethyl required a customs clearance to avoid the excise tax. Blood plasma was scarce and that which was available was committed to military use.^33^ Serum (plasma minus fibrinogen) was easier to obtain and the price more reasonable, although it was inferior because it lack the same capacity for coagulation.^34^ Among vaccines, the typhus vaccine was packed in 20 cc instead of the 30 cc initially requested, which was more standard.^35^ Tetanus toxoid could be supplied in either plain or the preferred alum precipitated form, which was easier to store and distribute. Despite its toxicity, the mercuric oxide was used as a preservative in medicines or as an antiseptic against bacterial infections and to remove mould, so it needed to be supplied in small amounts; hence, a quarter ounce was more appropriate than the one ounce initially requested. The typhoid vaccine was supplied as a triple-typhoid vaccine with *Bacillus typhosus, Bacillus paratyphosus A* and *Bacillus paratyphosus B*, which was commonly accepted in military usage. The need to protect overseas troops from paratyphoid fevers necessitated inoculation against *Bacillus paratyphosus A* and *Bacillus paratyphosus B*; the fact that these infections would undoubtedly appear, sooner or later, in large cantonments, made it prudent to inoculate the entire army and civilians as a preventative measure.^36^ These specifications and modifications were all derived from Soper’s review.

As predicted by the North-Strong Report, it proved difficult to secure sufficient medical supplies. Prices rose steeply in August once procurement began, largely in response to US military demand. Prices for all items had already increased by 10–30 per cent over the previous 2 months, and manufacturers were reluctant to quote unless a firm order was placed. With an urgent request from the UNC, and as the UN received cash for this purpose from the Korean Relief Assistance Fund, it transferred sums to the WHO.^37^ Most of the items were ready to ship within a few weeks, but some were scarce and depended on priority allocations from the US government.^38^

In November 1950, the battlefield situation changed dramatically when the Chinese army crossed the border, pushing UN forces back into the south. This rapid retreat complicated medical support for both military personnel and civilians.^39^ The cost of Unified Command Request No. 10, for medical supplies to Korea, was estimated at $15,487,416. It comprised 119 items (59 drugs and 60 dressings).^40^ John R. Murdock, acting director of the WHO’s regional office, handled this urgent request, facing challenges in acquiring sufficient funds and timely market purchases. He offered expertise in preparing Request No. 10, similar to the August list of 29 items.^41^ To meet urgent demand, the UN sought new sources of supply. According to the North-Strong Report, the British drug market was relatively stable compared to that in the USA, which was dominated by military demand. Some British companies had ample supplies and could meet the requirements from on-hand stock or within a period of 1–2 months.^42^

Paying close attention to certain types of medication provides insight into the functioning of medical supply provision and administration. Sulpha drugs, morphine hydrochloride, codeine phosphate and epinephrine solutions were among the key medicines displaying this predicament, demonstrating not just the entire procurement and delivery procedure but also the distinct aspects of each medicine. One of the most urgent needs identified by the UN was to secure the supply of sulpha drugs such as sulphathiazole, sulphadiazine and sulphaguanidine. These drugs were developed between the two world wars and used to prevent and treat a wide variety of infections.^43^ Even after the advent of penicillin and its widespread use by Allied forces from 1944, sulpha drugs were used extensively.^44^ As supplies of penicillin were still very limited in 1950, it was used sparingly and chiefly for sulphonamide-resistant infections.^45^ Resistance was often detected in sexually transmitted infections, for example.^46^ Because sulpha drugs were scarce in the US market, the UN contracted with British firms that offered lower prices, inclusive of all transport and other charges, than their US competitors.^47^ In particular, Burroughs Wellcome & Co., based in London, supplied sulphaguanidine tablets in packs of 1,000 at a rate of 2 million tablets per month for 22 shillings 9 pence ($3.19) per thousand tablets.^48^ The company was able to supply multiple products at more reasonable prices than those offered by US companies.^49^

At the onset of the Korean War, there was also an urgent need for specific medical products, particularly morphine hydrochloride, codeine phosphate and epinephrine solutions. Although intended for civilian relief, these medicines were considered essential in military contexts. Morphine, an opiate, had long been used medicinally.^50^ In both world wars, morphia was widely used as a painkiller and also sometimes to treat ‘hysteria’ caused by the bombardment of cities.^51^ Also derived from opium or morphine, codeine was used to treat pain, coughs and diarrhoea by binding to opioid receptors in the central nervous system. Specifically, codeine phosphate served as an opioid, analgesic, anti-tussive (anti-cough) and antidiarrheal agent. Like other narcotic painkillers, it was highly addictive and this was to be the source of some problems in Korea.^52^ Morphine hydrochloride and codeine phosphate were potent painkillers and had been used to control postoperative pain, which was regarded as essential for hostile situations. Epinephrine, known as adrenaline, is a hormone and neurotransmitter used to treat allergic reactions, restore cardiac rhythm and manage mucosal congestion, glaucoma and asthma.^53^ These three medicines were among the first requested at the outbreak of the Korean War.^54^

The total cost of the three drugs amounted to £8,177 (codeine phosphate, 280,000 tablets; morphine hydrochloride, 668,000 ampules; epinephrine, 334,000 ampules). However, the original budget allocated by the UN for these medicines was only £4,285, including packing, shipping and insurance. Because of the high costs, Brig.-Gen. Reginald Parminter, a former Belgian soldier and then a member of the UN Secretariat in New York, had to adjust the budget by either seeking cheaper suppliers or continuing with Burroughs Wellcome & Co.^55^ On 29 March, J. D. Sinclair, chief of the Supplies Service Section of the WHO in Geneva, contacted other UK and French suppliers to renew their offers for the three medicines. Sinclair visited the UK, contracting with suppliers and the Bank of England for detailed information and fund transfers to cover the French purchases. He proposed purchasing codeine phosphate from Burroughs Wellcome & Co. for £1,100, morphine hydrochloride from Evans’ Medical Supplies for £4,735 and epinephrine from Phamigia, France, for £1,525, bringing the total price down to £7,360.^56^

In April, British Drug Houses offered a better price for codeine phosphate, £910, lowering the total cost for the same amount of medicine to £7,170. The amount did not include insurance or freight. The order also included the purchase of ascorbic acid (vitamin C) for an additional £1,750 on the same order.^57^ Parminter had already spent £2,650 of the £5,600 deposited to the WHO London account on 15 January 1951, leaving £2,750 to cover the additional purchases. The WHO arranged for the transfer of £8,685 to its account with Lloyds and National Provincial Foreign Bank in London, which included the remaining Ethiopian contribution for Korean relief, making the purchase of these supplies possible.^58^ The total purchases amounted to £9,593, and when combined with previous purchases of ascorbic acid from Burroughs Wellcome & Co. for £2,850, the final expenditure reached £12,443.^59^ Regarding the certificates for codeine phosphate and morphine hydrochloride, Sinclair and Parminter had to coordinate with the Home Office.^60^ To sum up, medical supplies required a distinct supply chain and procedure for storing and distributing them because they needed to be obtained for specific civilian needs. A limited number of suppliers had to be screened in an unstable medical supply market. The medicines were shipped separately from London in June and arrived in Yokohama, Japan, in August and September, destined for Pusan (currently, Busan), Korea.^61^

### Finance and Logistics

Medical supplies under Unified Command Request No. 10 began arriving in Korea from March 1951, transported through multiple shipments and flights rather than a single trip. Prior to the arrival in Korea of UNKRA, the reception, transportation, warehousing, allocation, accounting and distribution of these materials was organised by the supply division of UNCACK. This involved working out mechanisms for payment, dealing with legal affairs and making precise arrangements for shipping and delivery.^62^

The logistical chain was complex because the relief effort had to rely a good deal on donations. As the WHO’s finances were limited, it was not in a position to supply medical items itself, and the UN had to appeal to various states for assistance.^63^ From November, donation offers were coordinated by John R. Murdock, the acting director of the WHO’s regional office. The Republic of China (Taiwan), Denmark, India, Israel, the Philippines, Turkey, the UK and Venezuela offered various medical supplies. Additionally, the International Refugee Organisation and the United Nations International Children’s Emergency Fund provided supplies.^64^

Payments and funds related to UN purchasing affairs were processed by UK banks. For example, for the initial portion of Unified Command Request No. 10, the UN secured funding from the Korean Relief Assistant Fund for medical supplies, transferring £5,600 to the WHO account at Lloyds and National Provincial Foreign Bank.^65^ The operating expenses of handling relief supplies throughout Korea amounted to over 1 million dollars per month, funded by the UN Special Account, primarily obtained from the sale of Civil Relief in Korea supplies in Korea.^66^

UNCACK’s supervision extended beyond the receipt of supplies, continuing until they were delivered to the end users. In this process, there were numerous legal issues that needed to be addressed, especially regarding narcotic drugs and bills of lading. Among the required medicines, codeine phosphate, morphine hydrochloride and tinctures of opium were controlled under the UK’s Dangerous Drugs Act.^67^ Therefore, to export them, the WHO required a certificate from the importing country (Korea) declaring that the import was within its drug quota. Thus, UNKRA prepared an import certificate form, signed by the Korean government, to ensure the WHO’s regional office and Burroughs Wellcome & Co. could export the drugs as needed.^68^ Copies of the certificate were sent to the narcotics section and suppliers, facilitating the shipment of these medicines.^69^

In addition, a detailed protocol for managing bills of lading was developed. One original bill was sent by registered airmail to the commanding general at the medical depot in Yokohama, Japan; another original and one copy were sent by registered airmail on a different flight to the commander-in-chief of the UNC in Tokyo, Japan; and a third original bill of lading was sent via ship’s bag. One copy of the bill was forwarded to Brigadier-General Parminter, coordinator of Korean Relief at the UN in New York, and another copy was sent to the WHO’s regional office in Washington, DC.^70^ For this shipment, the WHO informed the State Department in March that the UNC would receive certain medicines, including codeine phosphate, morphine hydrochloride and epinephrine.^71^ Narcotic painkillers and other sensitive medications were reported separately.

The journey of medical supplies was long and complex, with packaging being particularly crucial because of the warzone destination. The WHO mandated that the supplies ‘be packed in strong [and] seaworthy cases’ appropriate for export.^72^ Leslie Atkins, chief of the Supply Section of the WHO’s regional office, sent a letter to Brigadier-General Parminter, requesting exact marking instructions, including the consignee’s name, destination and the UN symbol on the packages.^73^ Full shipping details, such as gross and net weight, metric cubage, the name of the vessel and the date of shipment, were also required.^74^ UNCACK categorised aid goods into three types: Supply for Economic Cooperation (SEC), Supply for Korean Organization (SKO) and Sundry UN (SUN), each requiring a different accounting type. SEC included leftover supplies from the US Economic Cooperation Administration or Marshall Plan Programme before 1950. SKO comprised supplies acquired with allocated military funds to prevent famine, disease and unrest in Korea. SUN included supplies donated by UN member nations other than the USA, as well as philanthropic organisations in the USA and across the world.^75^ Most of the medical supplies initiated with purchase orders in August and November belong to the SKO and SUN.

Many companies were involved in supplying medical items, resulting in sequential shipments. On 7 February 1951, the WHO, on behalf of the UN, issued a purchase order (MS-583) to Burroughs Wellcome & Co., co-signed by the UN commanding general in Pusan.^76^ The agent of Turner & Co. Ltd. in London, responsible for handling this shipment, determined the most efficient shipping route from London to either Kobe or Yokohama, the two major ports in Japan. The WHO opted to transfer the supplies to the Office of Supply in Yokohama, a seaport city south of Tokyo, before forwarding them to Pusan. The port of Yokohama not only served as a key transport base for military personnel but also as a logistical hub for UN forces, surpassing the port of Kobe (which traditionally had close connections with Pusan) in both tonnage tax revenue and the volume of international passenger and cargo traffic.^77^

### Distributing to Korean Civilians

About 70 per cent of all relief supplies arrived in Pusan directly or through Japanese ports, the remainder being delivered to Incheon, Kunsan, Masan and Yosu. The UNC sought actively to monitor the unloading of civilian relief goods at the ports to avoid interfering with the rapid unloading and transhipment of military supplies. Upon arrival, ships’ manifests were compared to actual counts of items received by the Korean government, and the Supply Division of UNCACK notified the Korea Ministry and relevant UNCACK sections affected. The recommended allocations of supplies were based on the firm’s quarterly requirements and the country’s most pressing needs at the time of receipt. This allocation was determined through a conference between the relevant ministry and the UNCACK division. The proposed allocation was then presented at the weekly meeting of the Central Relief Committee, which was chaired by the minister of social affairs of the Government of Korea and included seven other ministers or their deputies, along with eight UNCACK members, including the commanding general and seven division and section chiefs. Once approved by the Central Relief Committee, the relevant department issued an allocation letter, which was forwarded to UNCACK’s movements and control section as well as the director of the Office of Supply in the Republic of Korea. Given the military priority for both rail and marine transfer routes, the chief of the movement and control section of UNCACK arranged for the necessary boxcars and ships. However, all physical handling of goods was carried out by Korean workers employed by the Office of Supply. UNCACK’s Office of Supply contracted with 14 warehouses to manage these goods.^78^

Warehousing companies were responsible for storing the supplies, whereas truck companies handled the transportation of supplies from the piers and unloading points to the warehouses. Forwarding companies were then arranged to move the goods from the warehouses by road or coastal shipping. From there, distribution was carried out using boats, motor coaches, rail cars, trucks and bullock carts. For urgent medical supplies, aeroplanes were also used.^79^ Sulphadiazine and penicillin from Mexico and boric acid ointment from Israel were transported by sea. However, many medical supplies, including vaccines (cholera vaccine, typhus vaccine, typhoid vaccine, diphtheria vaccine, smallpox vaccine, tetanus antitoxin, etc.) and blood, were transported by air to Pusan, Kimpo, Wonsan, Taegu and Taejon. As well as responding to the urgent need for such products, this allowed for the refrigeration necessary to preserve them.^80^ Transporting supplies from ports in smaller towns and cities posed a significant challenge due to the military demands on rail transportation. In April 1951, an average of only 3.7 rail cars per day were available for moving supplies inland from Pusan. During the same period, approximately 10,000 metric tons were received at this port, creating an average need for 17 cars per day. The insufficient inland transportation resulted in Pusan warehouses being overwhelmed, with an estimated 80,000 tons of relief supplies, including medical items.^81^

The Office of Supply, under UNCACK supervision and in compliance with Central Relief Committee allocations, distributed these supplies to provinces. With support from the Provincial Relief Committee, distribution continued from the provincial level down to the *gun* or county level. The provisions were eventually dispersed to numerous *myuns* (small villages). UNCACK field personnel strictly monitored the delivery of all humanitarian goods within the provinces,^82^ for it was critical for the UN to supply and distribute medical supplies to specific localities according to need. To do this effectively, the UN had to coordinate civilian aid with the USA-led UNC, particularly while the battle continued and armistices postponed. Although UNCACK (under the auspices of the UNC) led the distribution of medical supplies in South Korea near the battlefield, the UN handled the procurement and delivery of the supplies, as well as the administrative processes, through the WHO, which was later transferred to UNKRA.^83^ Thus, the UN established a viable framework for civilian aid during the Korean War, which would be expanded in the coming decades.

However, the supply and distribution of medicines in the field did not always go as planned, particularly with regard to morphine hydrochloride. On 16 September 1952, while UNKRA was gradually taking over responsibilities, the agent-general received a telegram from the commander-in-chief of the UNC regarding a surplus of morphine hydrochloride purchased in 1951. According to purchase orders MS-583 and MS-637, Burroughs Wellcome & Co. supplied 11,400 ampules of 20 mg and 99,960 of 10 mg, while Evans Medical Supplies Ltd. supplied 668,000 ampules of 10 mgm.^84^ UNKRA notified the WHO about the surplus and proposed exchanging it for other items needed for the Korean Emergency Relief Programme.^85^ In 1951, a total of 762,800 ampules of morphine hydrochloride were acquired between 30 January and 9 April. In the same year, 2,000 ampules were provided to 380 basic medical units. In 1952, the amount of morphine hydrochloride was reduced to 100 ampules per basic medical unit. Despite this reduction, stocks in Korea were cancelled during the second, third and fourth quarters of 1952. In 1953, the allocation of 54,000 ampules was planned, leaving 702,780 ampules stored at the Medical Depot in Japan, which would cover enough for 13 years of use. The UNC requested advice on what to do with the 648,780 ampules of morphine hydrochloride, noting that ‘this quantity of narcotic drug would cause serious problems.’^86^

The UNC was ‘anxious to move this drug from the Far East’ because of the challenges of narcotic control and warehousing. The USA sought to resolve the issue by asking whether the WHO could use the surplus morphine hydrochloride in another programme or if the original suppliers (Evans Medical Supplies Ltd. and Burroughs Wellcome & Co.) could restock or dispose of it in the British market.^87^ J. D. Sinclair, chief of the supply services section of the WHO, had led the original trade in 1951 and mediated with two companies. To initiate negotiations, Sinclair requested a detailed statement showing the quantities procured from each supplier, the quantitative value of the ampules and their condition, given that morphine solutions are unstable when exposed to light or temperature changes.^88^ The UNC provided additional information, confirming that all of the morphine hydrochloride ampules were in good condition.^89^ At the same time, ‘a more recent list of medical requirements for Korean Relief’ was required because of shifting priorities, and efforts to solve the issue lasted until 1953.^90^

In January 1953, Evans Medical Supplies Ltd. planned to dispose of most of the surplus ampules in Egypt. The Egyptian government had issued a tender for 3,000 boxes of 12 ampules, totalling 36,000 ampules, and it was preparing to issue another tender for 16,000 boxes of 6 ampules, totalling 96,000 ampules, bringing the grand total to 132,000 ampules. Given the lack of offers for these surplus drugs up until that point, it was decided that the best approach would be to leave the pricing decision to Evans Medical Supplies Ltd., a reputable firm that would act in the best interest of the Unified Command.^91^ However, in February 1953, an Egyptian agent of Evans Medical Supplies Ltd., which was a UK corporation supplying morphine ampules for the WHO, sent a letter to arrange the purchase of the drug. The Egyptian government had changed its drug policy, abolishing all licences, which necessitated finding additional buyers and exploring other options.^92^

At the time, opiate and morphine addiction was a major issue within the UN forces and South Korean society, though it often went unnoticed during the war. Several cases of morphine supplies falling into the wrong hands were reported, including surplus war stocks containing morphine being sold to the public and thefts from several medical depots, though these cases likely involved only small amounts. In addition, because of the scarcity of qualified workers at first aid posts and mobile units, it was crucial that all volunteer assistants be trained to administer morphine. This situation led to an expansion of the circle of individuals responsible for administering narcotic painkillers, including morphine, which in some cases resulted in the loss or theft of these medications—a potential major scandal, particularly in remote, rural areas.^93^ Remarkably, the prospect of a Chinese opium conspiracy loomed large in US drug circles, with suspicions that Communist China sought monetary gain by increasing the opium threat to US troops in Korea.^94^

The UN’s efforts in the procurement and distribution of medicines were not always successful. One reason for this was the shortage of funds for purchases, which led the UN to rely heavily on assistance and donations from member nations. As a newly formed organisation, the WHO had limited administrative capabilities and lacked the funding necessary to manage these tasks. It struggled to accurately predict and manage demand, particularly for narcotic analgesics. This was the challenging situation the UN faced in the early 1950s.

### Supplying Health-Care in the Field

In 1952, UNKRA arrived in Korea and gradually began its mission, which included planning a rehabilitation programme, hiring medical experts and procuring necessary supplies to rebuild social and economic functions in Korea.^95^ Before its first move, UNKRA requested that the UN’s specialised organisations survey their respective areas to establish rehabilitation programmes. Among these planning missions, the WHO/UNKRA mission visited Pusan, Korea, on 19 August 1952 to investigate the medical and health situation and develop a long-term plan for rehabilitating Korea’s medical system.^96^ The WHO team cooperated closely with UNCACK’s medical division, receiving a briefing on Korea’s public health and medical services and presenting their planning report to UNKRA and related organisations. When the WHO dispatched a health mission to the Korean peninsula in 1952 in cooperation with UNKRA, three of the commissioners, including the mission’s leader, Professor George MacDonald, contributed their UK experience to the commission’s report. MacDonald was an expert in tropical medicine, being particularly known for his work on malaria. He had worked in India and Sierra Leone as well as serving in a variety of locations with the British Army during the Second World War.^97^ Along with UNKRA’s Secretary-General, Sir Arthur Rucker, a former civil servant in Britain’s Ministry of Health, who had left for Geneva to direct the International Refugee Organization, his experience was instrumental in shaping UNKRA’s approach.^98^

One of their most important duties was to plan the allocation of medical supplies. Their allocation was vital to the functioning of the entire system of medical relief, from large hospitals down to mobile clinics. The WHO’s report made many recommendations relating to the efficient distribution of medical supplies and the development of a system for civilian relief and subsequent rehabilitation. Given the predominantly rural nature of the country, it was deemed cost-effective and desirable to establish a number of small medical facilities capable of providing out-patient care in addition to hospitals. In addressing public health and curative activities separately, the aim was to emphasise that while the same building and some staff might be used for both objectives, the activities should be carried out independently. Any attempt to combine preventative and curative work would likely fail under the medical conditions in Korea, where conflict was ongoing. The curative functions of health units as first aid posts included examining and treating minor ailments and referring serious cases to hospitals. Patients were to be admitted based on the Health Unit medical officer’s recommendation. The recommended minimum staff was a medical officer, one nurse and two assistant nurses. The officers paid with Korean government funds were all temporary at the time, which could explain the poor quality of work. It would, however, make it easier to terminate the services of officials who did not perform well. No medical officer in charge of a unit had even completed a basic course of postgraduate training in public health. The nurses were relatively young and had not received any medical or nursing training; otherwise, they had just completed their studies.^99^ Special clinics for tuberculosis and venereal diseases were also to be established, with fixed consultation days. Mobile clinics, also known as mobile dispensaries, would bridge the gap between health units and hospitals. These were considered ‘a useful asset in the early stages’, when there was no lodging or manpower to serve ‘the more remote, isolated areas.’^100^ For efficiency, these clinics needed to visit specific places on set days. The medical system could be reinforced by mobile clinics, which were initially deployed in two provinces with plans for expansion if proven effective.^101^

When the Second World War broke out, the UK possessed a layered system of casualty evacuation for civilians which was synchronised with medical supplies. This was based on the evacuation chains that had been used during the First World War and some other conflicts. Casualties were managed using a military-style three-tiered chain of evacuation: first aid posts, mobile units and fixed or semi-fixed hospitals. Injuries were primarily classified into two categories: severe (including fatalities) and minor. The first aid posts, staffed by trained doctors, treated walking wounded and those with minor injuries or shock. The treatment mainly involved cleaning wounds, applying dressings, providing warm drinks and offering reassurance. Mobile units, often modified trucks equipped with emergency medical supplies, responded to emergencies. These units comprised a doctor, nurse, two stretcher bearers, morphine supplies, a car and equipment for transporting injured personnel. Hospitals were reserved for the most critical cases requiring long-term treatment.^102^

UNKRA adapted this layered evacuation system—with which Rucker and MacDonald would have been very familiar—to Korea, proposing changes to out-patient health clinics in 1952 as the focus shifted from emergency relief to rehabilitation.^103^ Beyond the battle lines of the Korean War, mobile clinics were used for civilian aid in a collaborative effort between UNKRA, UNCACK and the Korean government. UNKRA procured and imported four mobile medical clinics to Korea.^104^ Louis Findlay, as a medical consultant, played a pivotal role in devising, executing and revising medical projects. UNKRA adjusted specifications, considering the more compact Picker Jeep Mounted Mobile Laboratory compared to the initially considered clinics. They believed that the solution to the problem of equipping additional clinics could be achieved by using vehicles like the Jeep, which had been used to evacuate military casualties during the Second World War and in the front-lines in Korea. Some UNKRA members, including Dr Findlay, considered using the British Land Rover, which had a slightly larger body and was similar to the Jeep.^105^ In addition, UNKRA made and executed all necessary arrangements for the transportation and delivery of these vehicles to the Port of Pusan as quickly as practicable and at no cost to the US government. UNCACK oversaw all operational aspects of the project before the Korean War ended. This included managing the operations of the mobile clinics and supplying and replenishing medical expendables and other related items as needed. The UNCACK field teams were tasked with providing housing and facilities for each clinic’s international staff and assisting in arranging accommodations for Korean staff, ensuring they received the same level of support as their indigenous counterparts.^106^ The Korean Ministry of Health was responsible for coordinating with the operational agency to ensure the smooth running of the mobile clinics. The minister of health oversaw the recruitment, salaries and travel expenses for Korean medical personnel, drivers and trainees, all of whom were paid from the Ministry’s dispensary team budget. All equipment and accessories were imported into Korea free of duties, customs and taxes.^107^ The total cost of the mobile clinic project was $132,112, covering vehicles, medical supplies and operational necessities such as fuel and personnel.^108^

The mobile clinic was one of the special projects that involved collaboration not only with UN-affiliated organisations but also with entities beyond the commercial market. This project represented an advanced stage of design, surpassing mere mechanical skill. In April 1952, Messrs Boura & Forgas Ltd., a London corporation, supplied specialised vehicles and trailers, including diesel alternators, diesel pumps, tractors and excavators, for the director of UNKRA in Geneva.^109^ Tours & Forgas Ltd., an associate company of Messrs, provided a brochure detailing the specifications of a typical mobile dental and medical clinic, which could be redesigned for medical and surgical use.^110^ It was designed and manufactured in collaboration with the chief designers of Coventry Steel Caravans and Messrs Brockhurst. Typical designs comprised refurbished 4 × 4 Ford or Chevrolet XWD trucks with diesel alternators positioned in the back. These robust ex-army vehicles were considered the most suitable for the terrains over which the units would travel. They also provided a 3-ton lorry for carrying general stores, with special facilities for off-loading the diesel alternator.^111^ Heating was included, and if air cooling and conditioning were necessary, a roof-mounted unit could be installed at a cost of about 250 pounds per clinic. The alternators supplied were generally sufficient to meet these needs without additional power supply. A specific wall structure with high thermal insulation was required for the caravan. The caravan shells and chassis were of unique design, providing ‘proper working conditions, extreme robustness, minimum maintenance and an almost unlimited life’.^112^ The same type of caravan shell was delivered to the United Nations Relief and Works Agency for Palestinian refugees, along with a restored Ford 4 × 4 towing vehicle and diesel alternators.^113^ The mobile clinics were designed to be robust and adaptable, providing the necessary mobility for effective emergency relief operations.

The medical clinics had to be equipped with all necessary supplies, including medicines and equipment, to address various medical conditions in remote areas. For example, the Chungchong Namdo team followed a preliminary schedule that included a 5-day field trip, 1 day for vehicle maintenance and medical supply replenishment and 1 day for crew relaxation. Areas of operation were announced in advance to maximise patient care without wasting time. It was predicted that 100–200 people would receive treatment each day. The vehicle amenities also served as living quarters and provided a place to prepare meals in regions where such essentials were scarce.^114^ According to the specifications, Picker International provided equipment such as air conditioning, heating, lighting, storage cabinets, a surgical environment and a curative capacity equivalent to that of a semi-hospital. This included more advanced medical equipment like a biological refrigerator, microscope with two eyepieces, a condenser, transformer, lamp, centrifuge and distilling machine.^115^ The mobile units also came in 1-, 2- and 3-inch bandage gauze sizes, each of which included two dozen, two 4 oz aseptic syringes; six bottles for urine; one set of all-purpose laboratory reagent kit; and a set of otoscope and ophthalmoscopes. There was a specially compiled list of medications for mobile units for various situations. Because of limited space and specific objectives, the quantities and dosages of medicines were calculated and stocked in units.^116^ Louis Findlay persuaded Rucker that mobile clinics could be designed for various purposes and functions, including general medical, minor or major surgical, maternal and child health, diagnostic and X-ray services, tailored to Korean conditions.^117^

The mobile medical units patrolled the provinces to treat potentially fatal illnesses, respond to emergencies and manage epidemic outbreaks. These clinics, set up on a standard 1-ton Chevrolet truck, offered general practice, light surgery and immunisation services.^118^ They were equipped with medicines and equipment based on a layered system. More importantly, the mobile clinics were intended to meet both the immediate and long-term medical needs in Korea. UNKRA also devised the mobile clinic project ‘to improve the public health standard in Korea’; to provide medical services, environmental information and education in ‘remote and heretofore inaccessible areas’; to train Korean personnel to operate such clinics and enhance their medical skills and to determine the feasibility of expanding these units in the future as part of a long-term, integrated public health programme.^119^ UNKRA’s agent-general revised the project details to include education purposes. These revisions required design changes, including the addition of a public address system, projection equipment and a daylight screen at the back of the vehicles, double doors that opened outward with an outside awning stop and a Bell and Howell 16 mm projector with focal length lenses.^120^ The value of this approach was demonstrated in an experiment in Chungchong Namdo where a truck converted into a mobile clinic travelled to locations without accessible medical facilities, provided essential treatments efficiently and serving as a teaching post for Korean personnel.^121^

## Conclusion

During the Korean War, medical personnel faced severe shortages of supplies and equipment while confronting infectious diseases and other health risks among civilians. Providing essential equipment and medications became a top priority. Several preparatory steps were necessary to ensure a steady supply of medical materials. The 1950 international medical market required the establishment of a reliable supply chain for vital materials. Unlike most military operations directly led by the USA, the UN-coordinated civilian relief efforts by developing a comprehensive logistical system, legal framework, payment mechanism using UK banks and special care for some sensitive drugs, such as opioids. Medical and supply specialists from WHO’s regional office in Washington, DC, were involved in the procurement process. The effort to manage the distribution of medical supplies also laid the foundation for the future hospital system in Korea.

Soon after the war ended in armistice, the Korean government needed assistance with procurement and distribution during reconstruction. In October 1953, after just 3 months the Korean War ended in armistice, hearings were held in the US Embassy in Seoul before a subcommittee of the agency on government operations in the 83rd Congress. Participants in the hearings acknowledged intensive campaigns against health and medical problems produced ‘dramatic effects in the reduction of disease’. In July 1953, the US Congress already approved additional funds to accelerate reconstruction and rehabilitation efforts in Korea.^122^ UNKRA collaborated with Korean medical corporations and government agencies to plan local pharmaceutical supply, manufacturing and processing. This included discussions about free drug importation and government support for manufacturing approximately 100–110 critical drugs from a list created by the ministry.^123^ As the hospital system developed, both government-sponsored institutions and the private sector required equipment and supplies while the public demanded basic products. A cooperative distribution system between medical associations and companies was envisioned, along with the costing and accounting of supplies to government institutions. Over time, the demand for aid was expected to decrease.

In sum, during the Korean War, a system for delivering civil medical assistance was developed based on older military templates, alongside an entirely new system for obtaining and distributing medical supplies. This system operated independently from the US military demands to maximise the use of international suppliers and avoid market inflation. This article has elucidated the key features of this system and, in so doing, has highlighted the importance of medical logistics more generally. In 1954, after the war ended, Rucker insisted that ‘the restoration of Korea is as much a part of the purpose of the United Nations as was the fight against aggression’.^124^ Unless due consideration is given to the supply of medical items, it is impossible to fully appreciate the operation of civil or any other kind of medical assistance in the field.

## Footnotes

1 Leland M. Goodrich, *Korea: A Study of US Policy in the United Nations* (Westport: Greenwood Press, 1979), 128–33.

2 This study focuses exclusively on United Nations-supported South Korea, also known as the Republic of Korea. References to Korea in the text pertain to South Korea or the Republic of Korea unless specified otherwise.

3 Terrence J. Gough, *U.S. Army Mobilisation and Logistics in the Korean War: A Research Approach* (Washington, DC: Center of Military History United States Army, 1987), Preface; Janet MacDonald, *Supplying the British Army in the Second World War* (Barnsley, UK: Pen & Sword, 2020).

4 Jeffrey L. Arnold, ‘International Emergency Medicine and the Recent Development of Emergency Medicine Worldwide’, *Annals of Emergency Medicine*, 1999, 33, 97–103; Maged Dessouky et al., ‘Rapid Distribution of Medical Supplies’, in Randolph Hall, ed, *Patient Flow: Reducing Delay in Healthcare Delivery* (Berlin: Springer, 2006), 309–38.

5 Dong Choon Kim, ‘Forgotten War, Forgotten Massacres: The Korean War (195–1953) as Licensed Mass Killings’, *Journal of Genocide Research*, 2004, 6, 523–44; Heonik Kwon, ‘Korean War Mass Graves’, in Francisco Ferrandiz and Antonius C. G. M. Robben, eds, *Necropolitcs: Mass Graves and Exhumations in the Age of Human Rights* (Pennsylvania: University of Pennsylvania Press, 2015).

6 UNRRA evolved into a major purchasing agency, a global shipping network and a sophisticated medical emergency organisation covering over a dozen European countries with a staff of 10,000 trained personnel. William I. Hitchcock, *The Bitter Road to Freedom: A New History of the Liberation of Europe* (New York: Free Press, 2009), Ch. 6; Grace Fox, ‘The Origins of UNRRA’, *Political Science Quarterly*, 1950, 65, 561–84.

7 Jessica Reinisch, ‘Auntie UNRRA at the Crossroads’, *Past and Present*, 2013, 8, 70–1.

8 Jessica Reinisch, ‘Internationalism in Relief: The Birth (and Death) of UNRRA’, *Past and Present*, 2011, 6, 259–60; idem, ‘Introduction: Relief in the Aftermath of War’, *Journal of Contemporary History*, Special Issue on ‘Relief in the Aftermath of War’, 2008, 43, 371–404.

9 G. Daniel Cohen, ‘Between Relief and Politics: Refugee Humanitarianism in Occupied Germany, 1945–1946’, *Journal of Contemporary History*, 2008, 43, 437–49; Joseph E. Davies, *A Report to the President by the President’s War Relief Control Board* (Washington, DC: United States Government Printing Office, 1946), Preface; James H. George, ‘Another Chance: Herbert Hoover and World War II Relief’, *Diplomatic History*, 1992, 16, 389–407.

10 Beyond the relief efforts for immediate needs discussed in this article, UNKRA funds were used for reconstruction. With this help, mines and factories were reconstructed, agricultural and fishery industries were revived, communications were fixed and social services were rehabilitated to the greatest extent feasible. Arthur Rucker, ‘Korea – The Next Stage’, *International Affairs*, 1954, 30, 313–9; Gene M. Lyons, ‘American Policy and the United Nations’ Program for Korean Reconstruction’, *International Organisation*, 1958, 12, 180–92; Ryan Patrick Francis McMahon, ‘Unfinished, Unloved, UNKRA: The Formation, Life and Financial Enervation of the United Nations Korean Reconstruction Agency (1950–1954)’, PhD Dissertation, The Ohio State University, 2017, 20.

11 ’UNC-UNKRA Agreement’, 1951, S-0526-0337-0001, The United Nations Archives and Records Management Section (hereafter UNARMS); McMahon, ‘Unfinished, Unloved, UNKRA’, Introduction.

12 From 10 July 1951 to 27 July 1953, the Armistice Talks between the UN forces and the Communist forces aimed at peaceful resolution of the Korean War. The Soviet Union suggested the truce; the USA agreed to it; the commanders of the UN forces, North Korean army and Chinese army signed the agreement therefore ending the Korean War. The South Korean delegate rejected the armistice and did not sign it until the end. William Stueck, *The Korean War: An International History* (Princeton: Princeton University Press, 1995), Ch. 6 and 9.

13 The UNC was based in Tokyo, where the commander-in-chief received advice on all civil matters from his G-5 staff (the civil-military operations office at the division level). In Korea, civil assistance responsibilities fell to the commanding general of the Communication Zone (General Thomas Herren), who was supported by his G-5 staff in Pusan. He managed operation through the UNCACK, with his own headquarter of the Civil Assistance Command located in Pusan. ‘Notes on the Tasks Assigned to the Team’, 4 August 1952, S-0526-0337-0001, UNARMS.

14 WHO/UNKRA, ‘Health Planning Mission in Korea, WHO Survey’, UNKRA, 1952, 3–4; Lisa M. Brady, ‘Sowing War, Reaping Peace: United Nations Resource Development Programs in the Republic of Korea, 1950–1953’, *The Journal of Asian Studies*, 2018, 72, 351–63.

15 USA, ‘Relief and Rehabilitation in Korea: Hearings before a Subcommittee of the Committee on Government Operations, House of Representatives, Eighty-third Congress, Second Session’, 13, 14 and 16 October 1953 (Washington, DC: US Government Print Office, 1954), 15 and 17–8.

16 James A. Huston, *The Sinews of War: Army Logistics, 1775–1953* (Washington, DC: Government Printing Office, 1966); Marvin A. Kreidberg and Merton G. Henry, *History of Military Mobilisation in the United States Army, 1775–1945* (Forest Grove: University Press of the Pacific, 2005); Roderick L. Vawter, *Industrial Mobilisation: The Relevant History* (Forest Grove: University Press of the Pacific, 1983).

17 Terrence J. Gough, *U.S. Army Mobilisation and Logistics in the Korean War*; Michael Kendall, ‘An Inflexible Response: United States Army Manpower Mobilisation Policies, 1945–1957’, PhD dissertation (Duke University, 1982); James A. Huston, *Guns and Butter, Powder and Rice: U.S. Army Logistics in the Korean War* (Selinsgrove: Susquehanna University Press, 1989); Charles R. Shrader, *Communist Logistics in the Korean War* (Westport: Greenwood Press, 1995).

18 For example, Youngsoo Kim, ‘The United Nation’s Civil Assistance Command in Korea’s (UNCACK) Public Health Measures on Koje Island during the Korean War’, *Korean Journal of Medical History*, 2023, 32, 931–966; Dongwon Lee, ‘United States-United Nations Relations in the Korean War: Focusing on the Conflict over Aid Operations and War Expenses’, *Korea Journal*, 2020, 60, 98–125; Im Ha Lee, ‘The Chronic Communicable Disease Control of the United Nation Civil Assistance Command, Korea (UNCACK)’, *The Historical Journal*, 2014, 281, 281–311.

19 Roberts Barnes, ‘Chief Administrator or “Political Moderator” Dumbarton Oaks, the Secretary-General and the Korean War’, *Journal of Contemporary History*, 2019, 54, 347–67; Kyu-Hwan Shin, ‘The Korean Pharmaceutical Industry and the Expansion of the General Pharmaceuticals Market in the 1950–1960s’, *Korean Journal of Medical History*, 2015, 24, 749–82; Thomas G. Weiss, David P. Forcythe and Roger A. Coate, *The United Nations and Changing World* (Boulder: Westview Press, 2011), 12–5.

20 There were multiple contributor organisations: the US Economic Cooperation Administration, Civil Relief in Korea (CRIK), UNCACK, UNKRA. The total amount of relief aid received from the USA and the UN member nations during the war was $471.9 million. The current value is about $3,973 billion, according to date from South Korea’s Ministry of Foreign Affairs. Young-Jo Yang, ‘UN Member Nations Support during the Korean War: Emphasis on Supply Support’, *Military History*, 2013, 87, 53–6

21 For example, see Sunil S. Amrith, *Decolonising International Health: India and Southeast Asia, 1930–65* (London: Palgrave Macmillan, 2006); Nandini Bhattacharya, *Disparate Remedies: Making Medicines in Modern India* (Montreal: McGill-Queens University Press, 2023); James L. A. Webb, *The Long Struggle against Malaria in Tropical Africa* (New York: Cambridge University, 2014).

22 Jaegoo Kang and Yunjae Park, ‘Unstable Expansion: The Development of the Military Medical Evacuation Chain in Korea’; Mark Harrison ‘Casualty Evacuation in Korea, 1950–53: The British Experience’; Sekwon Jeong, You-ki Min and Sangduk Lee, ‘Medical Support Provided by the UN’s Scandinavian Allies during the Korean War’, Special Issues on Medicine and the Korean War, *Korean Journal of Medical History*, 2024, 32.

23 ’Brock Chisholm to Trygve Lie’, 17 January 1951, S-0526-0029-0003, UNARMS; James Barros, *Trygve Lie and the Cold War: The UN Secretary-General Pursues Peace, 1946–1953* (DeKalb: Northern Illinois University Press, 1989); Robert Barnes, *The US, the UN and the Korean War: Communism in the Far East and the American Struggle for Hegemony in the Cold War* (London, UK: Bloomsbury Academic, 2014).

24 ’Telegram from Trygve Lie to Fred L. Soper’, 14 August 1950, S-0526-0039-0006, UNARMS; Robert Barnes, ‘Chief Administrator or Political “Moderator”? Dumbarton Oaks, the Secretary-General and the Korean War’, *Journal of Contemporary History*, 2019, 54, 360.

25 ’Samuel Reader to Fred L. Soper’, 14 August 1950, S-0526-0039-0006, UNARMS.

26 ’North-Strong Reports’, 14 August 1950, S-0526-0039-0006, UNARMS.

27 ’North-Strong Reports’.

28 ’North-Strong Reports’; Gough, *U.S. Army Mobilisation and Logistics in the Korean War*, 76–82.

29 29 medical items estimated at$4,122,500 and a gross weight of 1.5 million lb. The price included inland freight of$80,000 and export packing of$125,000. The price could have gone higher. ‘Andrew W. Cordier to Benjamin J. Brown’, 14 August 1950; ‘Telegram from P. L Soper to Trygve Lie’, 14 August 1950, S-0526-0039-0006, UNARMS.

30 The medical supplies were a part of the main request, including dispatch of health and hygiene professionals and supplies of cereals, candy, salt and general supplies. Yang, ‘UN Member Nations Support during the Korean War’, 59.

31 Yang, ‘UN Member Nations Support during the Korean War’, 57.

32 Each medical team would do public health work for Korea’s civilian population, consisting of one medical officer, one public welfare officer, one sanitation officer and one administrative officer capable of overseeing the team’s commissariat and transportation arrangements. ‘Trygve Lie to Director General UNISANTE’, Geneva, 5 August 1950, S-0526-0029-0003, UNARMS; Im-ha Lee, ‘The Population Control during the Korean War: Focus on the Document of the United Nations Civil Assistance Command, Korea (UNCACK)’, *Salim*, 2009, 33, 39–75.

33 ’Medical Supplies for Relief in Korea’, S-0526-0039-0006, UNARMS.

34 The advantage of whole blood over plasma was proved during World War II, but division medical installations employed blood plasma to prevent and cure shock. Despite the risk of transmitting the virus known as ‘serum hepatitis’ or ‘homologous serum jaundice’, other options were not available at the time. Only dextran and serum albumin would be substitutes. Whole blood in large quantities would later be used in Korea. Douglas Blair Kendrick, *Blood Program in World War II* (Washington, DC: Office of the Surgeon General, Department of the Army, 1964); Robert M. Hall, ‘Treatment of the Wounded at Chipyong-ni’, *Military Medicine*, 1998, 163, 127–32.

35 Vaccines include all or part of the target pathogen, sometimes alive, sometimes killed or weakened. Thus, it was necessary to provide exact doses and avoid sharing medications to prevent secondary infections from injections. Elena Conis, *Vaccine Nation: Changing Relationship with Immunization* (Chicago: The University of Chicago Press, 2015), 255–6.

36 Since the beginning of the war, the UNC had been managing communicable diseases through UNCACK. Biweekly reports from UNCACK show that infectious diseases such as smallpox, typhus and typhoid had been increasing since 1951. Lee, ‘Public Health and Sanitation’, 333–4; On inoculation, see: Christoph Gradmann, Mark Harrison and Anne Rasmussen, ‘Typhoid and the Military in the Early Twentieth Century’, *Clinical Infectious Diseases*, 2019, 69, S385–7.

37 ’Memorandum from Milton P. Siegel to H. C. Grant’, 20 September 1950, S-0526-0039-0006, UNARMS.

38 ’Fred L. Soper to Trygve Lie’, 18 August 1950, S-0526-0039-0006, UNARMS.

39 Jian Chen, *China’s Road to the Korean War* (New York: Columbia University Press, 1994); Jian Chen, ‘China’s Changing Aims during the Korean War, 1950–1951’, *The Journal of American-East Asian Relations*, 1992, 1, 8–41; Hao Yufan and Zhai Zhihai, ‘China’s Decision to Enter the Korean War: History Revisited’, *The China Quarterly*, 2009; Allen Whiting, *China Crosses the Yalu: The Decision to Enter the Korean War* (Stanford: Stanford University Press, 1960).

40 The number of items was 120, with one duplicate (Item 82, 83: Needles size 10, 3/8 circle). ‘Medical Requirements for Korea’, Unified Command Request No. 10, 15 November 1950; ‘Special Assistant for Korea to Harrison Lewis’, 6 December 1950, S-0526-0039-0006, UNARMS.

41 ’John R. Murdock to General Parminter’, 20 November 1950, S-0526-0039-0006, UNARMS.

42 ’Leslie Atkins to Brigadier General Parminter’, 21 December 1950, S-0526-0039-0006, UNARMS.

43 Sulpha drugs, initially invented as Prontocil, were developed in the mid-1930s and were widely utilised during World War II, alongside penicillin, which was later introduced. Thomas Hager, *The Demon under the Microscope: From Battlefield Hospitals to Nazi Labs, One Doctor’s Heroic Search for the World’s First Miracle Drug* (New York: Crown, 2007), Introduction; John E. Lesch, *The First Miracle Drugs: How the Sulfa Drugs Transformed Medicine* (New York: Oxford University Press, 2006), 3–4.

44 Peter Neushul, ‘Science, Government and the Mass Production of Penicillin’, *Journal of the History of Medicine and Allied Sciences*, 1993, 38, 371–95; Peter Neushul, ‘Fighting Research: Army Participation in the Clinical Testing and Mass Production of Penicillin during the Second World War’, in R. Cooter, M. Harrison and S. Sturdy, eds, *War, Medicine and Modernity* (Stroud, UK: Sutton Publishing, 1998), 203–24.

45 As antibacterial agent, sulpha drugs were very useful at the time. Especially, sulphaguanidine was a hygienic choice not only in military use but also in civilian settings for dysentery. Lesch, *The First Miracle Drugs*, 210 and 225.

46 K. Meghan Fitzpatrick, ‘Prostitutes, Penicillin and Prophylaxis: Fighting Venereal Disease in the Commonwealth Division during the Korean War, 1950–1953’, *Social History of Medicine*, 2015, 28, 555–75; Milton Lewis, *Thorns on the Rose: The History of Sexually Transmitted Diseases in Australia in International Perspective* (Australian Government Publish Service, 1998), 242. On clinical trials in World War II, see Mark Harrison, *Medicine and Victory: British Military Medicine in the Second World War* (Oxford: Oxford University Press, 2004), 156–8.

47 ’North-Strong Reports’; ‘John R. Murdock to Secretary-General’, 19 September 1950; ‘Letter from Special Assistant for Korea to Dr Soper’, 11 November 1950, S-0526-0039-0006, UNARMS.

48 Burroughs Wellcome & Co. marketed the tablets under the ‘Tabloid’ trademark, and the term appeared in the UNKRA materials frequently. However, tablets are used in this study for modern comprehension. Roy Church and E. M. Tansey, *Burroughs Wellcome & Co.: Knowledge, Trust, Profit and the Transformation of the British Pharmaceutical Industry, 1880–1940* (Lancaster, UK: Crucible Books, 2007), Introduction; Roy Church, *Burroughs Wellcome in the USA and the Wellcome Trust: Pharmaceutical Innovation, Contested Organisational Cultures and the Triumph of Philanthropy* (Lancaster, UK: Carnegie Publishing, 2015), 2.

49 ’J. A. Santangelo, Export Sales Manager to Leslie Atkins’, 1 December 1950, S-0526-0039-0006, UNARMS.

50 Karolina Brook, Jessica Bennett and Sukumar P. Desai, ‘The Chemical History of Morphine: An 8000-Year Journey, from Resin to Denovo Synthesis’, *Journal of Anaesthesia History*, 2017, 3, 50–5; Barbara Hodgson, *In the Arms of Morpheus: The Tragic History of Morphine, Laudanum and Patent Medicine* (Richmond Hill, Canada: Firefly Books, 2001).

51 Hallam, *White Drug Cultures and Regulation in London*, 158–9.

52 John Collins, *Legalising the Drug Wars: A Regulatory History of UN Drug Control* (Cambridge, UK: Cambridge University Press, 2021), esp. Ch. 2 and 6.

53 Greer Arthur, ‘Epinephrine: A Short History’, *The Lancet-Respiratory Medicine*, 2015, 3, 350–1.

54 ’J. D. Sinclair to Brigadier General Parminter’, S-0526-0039-0006, UNARMS.

55 Although the US dollar is applied in the preceding part, the unit of money in the next sections is the British pound sterling, which was used for most purchases and transactions. Also, it adheres to the monetary units used in the UNKRA materials. ‘Parminter to J. D. Sinclair’, 20 March 1951, S-0526-0039-0006, UNARMS; For reference to the actual value of the contracts, the current value of the £8,177 is approximately £236,572. https://www.bankofengland.co.uk/monetary-policy/inflation/inflation-calculator

56 ’Letter from Th. Huber to Parminter’, 29 March 1951, S-0526-0039-0006, UNARMS.

57 ’J. D. Sinclair to Parminter’, 3 April 1951, S-0526-0039-0006, UNARMS; ‘J. D. Sinclair to Parminter’, 6 April 1951, S-0526-0039-0006, UNARMS.

58 ’J. D. Sinclair to Parminter’, 3 April 1951.

59 ’J. D. Sinclair to Parminter’, 6 April 1951, S-0526-0039-0006, UNARMS.

60 ’J. D. Sinclair to Parminter’, 3 April 1951; J. D. Sinclair to Parminter, 16 April 1951, S-0526-0039-0006, UNARMS.

61 ’Parminter to Harrison Lewis’, 6 June 1951; ‘Parminter to R. Asher’, 20 August 1951, S-0526-0039-0006, UNARMS.

62 Colonel Robert C. Ross, ‘Distribution of Relief Supplies in Korea’, Director of Supply, UNCACK, 9 April 1952, RG 331, SCAP, Economic & Scientific Section Director of Economic & Planning, General Subject Files, 1945–52, Box 7691: Briefing Conference on Republic of Korea for United Command Mission to ROK (2), National Archive and Records Administration (hereafter NARA).

63 ’P. Dorolle to Trygve Lie’, 31 October 1951, S-0526-0029-0003, UNARMS; ‘Reference Cable Misc. 59 from WHO with regard to WHO Financial Commitments Korean Emergency Relief Teams’, 15 January 1951, S-0526-0029-0003, UNARMS.

64 ’Parminter to N. C. Anderson: Transfer of Funds from Korean Relief Assistant Fund’, 5 January 1951; ‘List of Medical Offers Requiring Evaluation’, S-0526-0039-0006, UNARMS; ‘Letter from Special Assistant for Korea to John R. Murdock’, 24 November 1950, S-0526-0039-0006, UNARMS.

65 Because the payment was paid in pounds sterling, the WHO made inquiries in both the UK and France, where sterling could be used for the remaining quantities of the items. ‘J. D. Sinclair to Parminter: Korean Relief Supply – Unified Command Request No. 10’, 8 January 1951, S-0526-0039-0006, UNARMS; In terms of financial aspects, New York briefly served as the world’s financial hub after WWII until the Eurodollar market was established in the 1950s, when London took the lion’s share. Under English common law, the Bank of England could allow loosely regulated overseas markets to develop. Charles P. Kindleberger, *The Formation of Financial Centers: A Study in Comparative Economic History* (Princeton: Princeton University Press, 1974), 65; Ronen Palan, ‘International Financial Centers: The British-Empire, City-States and Commercially Oriented Politics’, *Theoretical Inquiries in Law* 2010, 11, 149–76.

66 Colonel Robert C. Ross, ‘Distribution of Relief Supplies in Korea’.

67 The Home Office was charged with implementing the Act to meet the obligations that Britain had agreed to when it ratified the International Opium Convention of 1921. W. B. McAllister, *Drug Diplomacy in the Twentieth Century: An International History* (London, UK: Routledge, 2000), 9–39; Christopher Hallam, *White Drug Cultures and Regulation in London, 1916–1960* (London: Palgrave Macmillan, 2018), esp. Ch. 7.

68 ’J. D. Sinclair to Parminter’, 17 January 1951; ‘Letter from Special Assistant for Korea to J. D. Sinclair’, 26 January 1951; Sae Sun Kim of the Korean Embassy handled the matter of the certificates, forwarding the Korean government to clear the shipment upon arrival in May 1951. ‘Sae Sun Kim to Parminter’, 3 May 1951, S-0526-0039-0006, UNARMS.

69 ’Letter from Special Assistant for Korea to J. D. Sinclair’, 8 February 1951, S-0526-0039-0006, UNARMS.

70 ’Korea Relief Supply-Unified Command Req. No. 10’, 23 February 1951; ‘Request No. 3S/1-Supplies for United Nations Korean Relief’, 21 May 1951, S-0526-0039-0006, UNARMS.

71 ’Note of Telephone Message from US Mission’, 19 March 1951, S-0526-0039-0006, UNARMS.

72 ’Annex to Purchase Order MS-583’, S-0526-0039-0006, UNARMS.

73 ’Leslie Atkins to Parminter’, 29 December 1950, S-0526-0039-0006, UNARMS.

74 The procurement of medical supplies was accompanied by an international transaction and shipping procedure, so it was critical to know what currency to pay in as well as what units to use for packaging and shipping. For example, pounds were used to weigh items, and metres were used to measure lengths. Mixing the units up was a typical mistake that required checking multiple times. ‘Letter from Special Assistant for Korea to J. D. Sinclair’, 19 January 1951; ‘Letter from Special Assistant for Korea to J. D. Sinclair’, 26 January 1951, S-0526-0039-0006, UNARMS.

75 Colonel Robert C. Ross, ‘Distribution of Relief Supplies in Korea’.

76 This order included various medicines from Unified Command request No. 10, such as vitamin K (1,400 boxes of 6 ampoules), codeine phosphate (1,000 bottles of 100 tablets), morphine hydrochloride (450 boxes of 12 injections and 12 boxes of 500 injections plus 8,330 boxes of 12 injections after 2 months), tincture opium (350 bottles of 500 cc), ascorbic acid (100 bottles of 500 tablets and 100 bottles of 1,000 tablets), digitalis leaf (1,400 bottles of 100 tablets plus 1,500 bottles of 100 tablets after one month), adrenaline solution (1,600 boxes of 12 ‘Wellcome’ brand injection, 1 cc) and phenobarbital (700 bottles of 100 tablets plus 8,000 bottles of 100 tablets after one month). ‘Purchase Order M5-583’, 7 February 1951, S-0526-0039-0006, UNARMS.

77 ’Letter from J. D. Sinclair, Request No. 3S/1-Korean Relief Supply-Shipments to Korea’, 19 February 1951, S-0526-0039-0006, UNARMS; Japan functioned in i role as a rear base for the supply and transit of soldiers and materials throughout the Korean War. Ishimaru Yasuzo, ‘The Korean War and Japanese Ports: Support for the UN Forces and Its Influences’, *NIDS Security Report*, 2017, 8, 55–70.

78 UNCACK encouraged one of the largest renowned storage corporations to build 80,000 square feet of warehouses near Pusan’s Pier #2. UNCACK’s real involvement consists mainly of providing the contractor with essential building supplies at the landed cost. The new warehouses built in 1952, complete with extensive rail spur connections, would cut the average truck turnaround to roughly a mile, a 50 per cent decrease in operational expenses. Colonel Robert C. Ross, ‘Distribution of Relief Supplies in Korea’; Yunmi Kim, ‘U.N. Military Command during Korean War and Busan Port: The Company Established a Marine Logistics System’, *Journal of North-East Asian Culture*, 2022, 70, 23–42; John P. Dimoia, ‘Reconfiguring Transport Infrastructure in Post-War Asia: Mapping South Korean Container Port, 1952–1978’, *History and Technology: An International Journal*, 2020, 36, 382–99.

79 Colonel Robert C. Ross, ‘Distribution of Relief Supplies in Korea’.

80 United Nations Civil Assistance Command, Korea, ‘Statistical Report of CRIK Supplies Received during Fiscal Year 1951’, 22 August 1951, Supply Records Section, RG 469 UNCAC – Korea, 1951, NARA.

81 ’Phasing of CRIK Supplies for 1st Quarter of Fiscal Year 1952’, 3 June 1951, RG 338, UNCACK, 1951, Box 18, Supply Report File No. 400, NARA.

82 Because the Korean government owned these things, it was far more concerned with theft and abuse. Losses from all causes averaged less than 8 per cent, ‘a record any similar military supply unit would be very proud to achieve’. Colonel Robert C. Ross, ‘Distribution of Relief Supplies in Korea’.

83 McMahon, ‘Unfinished, Unloved, UNKRA’, Conclusion.

84 ’Sinclair to Clyde L. Hyssong: Statement Showing Quantities of Morphine Hydrochloride Purchased for Korea-Request 38/1’, 20 October 1952, S-0526-0039-0006, UNARMS.

85 ’Letter: 450/3/01’, 30 September 1952, S-0526-0039-0006, UNARMS.

86 ’Letter from United States Mission to the United Nations’, 16 September 1952, S-0526-0039-0006, UNARMS.

87 ’Clyde L. Hyssong to J. D. Sinclair’, 30 September 1952, S-0526-0039-0006, UNARMS.

88 ’Sinclair to Clyde L. Hyssong: Statement Showing Quantities of Morphine Hydrochloride Purchased for Korea-Request 38/1’, 20 October 1952, S-0526-0039-0006, UNARMS.

89 Quantities of surpluses from respective suppliers were as follows: 15,288 of 1/3 grains and 24,060 of 1/6 grain from Burroughs Wellcome & Co. and 561,990 of 1/6 grain from Evans Medical Supply Ltd. ‘UN-2968/1368’, 12 January 1953, S-0526-0039-0006, UNARMS.

90 ’Clyde L. Hyssong to J. D. Sinclair’, 23 October 1952, S-0526-0039-0006, UNARMS.

91 ’Letter from F. P. Wilson to UNKRA’, 12 January 1953, S-0526-0039-0006, UNARMS.

92 The location of these drugs after 1953 cannot be determined using UNKRA resources. They may have remained in Yokohama and then been shipped somewhere, or they could have been delivered to UNKRA or South Korea. ‘Letter from T. J. Garner to Supplies Service Sections, World Health Organisation’, 9 February 1953; ’A. M. Idreos to T. J. Garner’, 9 February 1953, S-0526-0039-0006, UNARMS.

93 William B. McAllister, *Drug Diplomacy in the Twentieth Century* (London: Routledge, 1999); Ji-Young Park, ‘The War on “Red Drugs”: Anticommunism and Drug Policy in Republic of Korea, 1945–1960’, *Korean Journal of Medical History*, 2016, 25, 77–110; William O. Walker, *Opium and Foreign Policy: The Anglo-American Search for Order in Asia, 1912–1954* (Chapel Hill: University of North Carolina Press, 1991).

94 Collins, *Legalising the Drug Wars*, 141–2.

95 ’Notes on the Tasks Assigned to the Team’, 4 August 1952, S-0526-0337-0001, UNARMS; Su-Hyeon Hong, ‘Postwar Health Assistance of Korea Civil Assistance Command (KCAC) and the “Rehabilitation” of Public Health in Korea’, *History & the Boundaries*, 2023, 126, 81–118.

96 UNKRA, ‘WHO and FAO Expert Attend UNCURK Meeting’, 9 August 1952, S-0526-0337-0001, UNARMS; WHO/UNKRA, ‘Report of the WHO/UNKRA Health Planning Mission in Korea’, S-0526-0337-0001, UNARMS, 2–3.

97 All three of the WHO/UNKRA health planning mission had UK-based medical experience. George MacDonald was a professor of tropical hygiene at the University of London and the head of the Ross Institute of Tropical Hygiene. He served as an honorary consultant malariologist for the British War Office as well as on the WHO Expert Committees on Malaria and Environmental Sanitation. Walter Wickremesinghe earned a medical degree from the Ceylon Medical College before moving to London to get British credentials and then to Harvard University to complete his studies in 1926. William P. Forrest had his medical and public health training in the UK, with an emphasis on infectious and communicable illnesses. ‘Personal File of George MacDonald’, ‘Personal File of W. G. Wickremesinghe’ and ‘Personal File of William P. Forrest’, S-0526-0337-0001, UNARMS.

98 Rucker, ‘Korea – The Next Stage’, 313–9.

99 WHO/UNKRA, ‘Report of the WHO/UNKRA Health Planning Mission in Korea’, 31–2.

100 WHO/UNKRA, ‘Report of the WHO/UNKRA Health Planning Mission in Korea’, 78.

101 WHO/UNKRA, ‘Report of the WHO/UNKRA Health Planning Mission in Korea’, 10.

102 R. E. W. Fisher, ‘Air Raid Precautions: Casualty Clearing Organisation’, *British Medical Journal*, 1938, 2, 397–402; ‘Air-Raid Casualty Services’, *The British Medical Journal*, 1940, 2, 716–7.

103 ’Letter from Dr L. Findlay to UNKRA in DC’, 8 May 1952, S-0526-0027-0001, UNARMS; Contardo Vergani, ‘The Italian Mobile Surgical Units in the Great War: The Modernity of the Past’, *Updates in Surgery*, 2020, 72, 565–72.

104 ’Mobile Clinics (UNKRA Project 711-A)’, S-0526-0027-0002, UNARMS.

105 ’Letter from Dr L. Findlay to UNKRA in DC’.

106 ’Project Agreement, Mobile Clinics’, 8 August 1952, S-0526-0027-0002, UNARMS.

107 ’Project Agreement, Mobile Clinics’.

108 The numbers were initially approximate. ‘Mobile Clinics (UNKRA Project 711-A)’, S-0526-0027-0002, UNARMS.

109 ’S.R. Hinds to A. B. Borthwick’, 9 May 1952, S-0526-0027-0001, UNARMS.

110 ’A. B. Borthwick to Findlay’, 5 May 1952, S-0526-0027-0001, UNARMS.

111 ’A. B. Borthwick to Findlay’.

112 ’A. B. Borthwick to Findlay’.

113 ’A. B. Borthwick to Findlay’.

114 ’Mobile Dispensary’, 7 January 1952, S-0526-00027-0001, UNARMS.

115 ’Mobile Medical Coach Division: West India Commercial Company Ltd.’, S-0526-0027-0002, UNARMS.

116 ’Medicines & Equipment for the Mobile Dispensary’, S-0526-00027-0001, UNARMS.

117 ’Findlay to Arthur’, 12 March 1952, S-0526-00027-0001, UNARMS.

118 ’Mobile Dispensary’, 7 January 1952, S-0526-00027-0001, UNARMS.

119 ’Mobile Clinics (UNKRA Project 711-A)’, S-0526-0027-0002, UNARMS.

120 UNKRAGE GENEVA, ‘Radiogram’, 94 FFF Pusan 91, 17, 07042, S-0526-0027-0001, UNARMS.

121 In the case of Chungchung Namdo Province, for example, Civilian Supply Officer Major Lawson R. Beard designed the fittings and furnishings of the mobile clinic, and John T. Wilson, a member of the team, supervised construction after approval by the team’s public-health officer and the provincial public health chief. ‘Mobile Dispensary’, 7 January 1952, S-0526-0094-0006, UNARMS; ‘Mobile Clinics (UNKRA Project 711-A)’.

122 USA, ‘Relief and Rehabilitation in Korea’, 175 and 208–9; WHO/UNKRA, ‘Report of the WHO/UNKRA Health Planning Mission in Korea’, 97.

123 ’First By-Monthly Report on Day-to-Day Activities of the WHO/UNKRA Health Planning Mission to Korea’, 30 August 1952, S-0526-0337-0001, UNARMS.

124 Rucker, ‘Korea – The Next Stage’, 313–9.

